# Calcaneal Tendon Plasticity Following Gastrocnemius Muscle Injury in Rat

**DOI:** 10.3389/fphys.2019.01098

**Published:** 2019-09-03

**Authors:** Fabrício Reichert Barin, Ivo Vieira de Sousa Neto, Graciele Vieira Ramos, Alexander Szojka, Amanda Lima Ruivo, Carla Tatiana Mota Anflor, José David Hurtado Agualimpia, Allan Corrêa Domingues, Octávio Luiz Franco, Adetola B. Adesida, João Luiz Quaglioti Durigan, Rita de Cassia Marqueti

**Affiliations:** ^1^Faculdade de Ceilândia, Universidade de Brasilia, Brasilia, Brazil; ^2^Universidade Paulista, Brasilia, Brazil; ^3^Centro Universitário ICESP, Brasilia, Brazil; ^4^Division of Orthopaedic Surgery, University of Alberta, Edmonton, AB, Canada; ^5^Division of Surgical Research, University of Alberta, Edmonton, AB, Canada; ^6^Group of Experimental and Computational Mechanics, Universidade de Brasília, Brasília, Brazil; ^7^S-Inova Biotech, Universidade Catolica Dom Bosco, Campo Grande, Brazil; ^8^Centro de Análises Proteômicas e Bioquímicas, Universidade Católica de Brasília, Brasília, Brazil

**Keywords:** muscle-tendon interaction, tendon disorders, tenocyte, muscle damage, calcaneal tendon

## Abstract

Cross-talk between skeletal muscle and tendon is important for tissue homeostasis. Whereas the skeletal muscle response to tendon injury has been well-studied, to the best of our knowledge the tendon response to skeletal muscle injury has been neglected. Thus, we investigated calcaneal tendon extracellular matrix (ECM) remodeling after gastrocnemius muscle injury using a rat model. Wistar rats were randomly divided into four groups: control group (C; animals that were not exposed to muscle injury) and harvested at different time points post gastrocnemius muscle injury (3, 14, and 28 days) for gene expression, morphological, and biomechanical analyses. At 3 days post injury, we observed mRNA-level dysregulation of signaling pathways associated with collagen I accompanied with disrupted biomechanical properties. At 14 days post injury, we found reduced collagen content histologically accompanied by invasion of blood vessels into the tendon proper and an abundance of peritendinous sheath cells. Finally, at 28 days post injury, there were signs of recovery at the gene expression level including upregulation of transcription factors related to ECM synthesis, remodeling, and repair. At this time point, tendons also presented with increased peritendinous sheath cells, decreased adipose cells, higher Young’s modulus, and lower strain to failure compared to the uninjured controls and all post injury time points. In summary, we demonstrate that the calcaneal tendon undergoes extensive ECM remodeling in response to gastrocnemius muscle injury leading to altered functional properties in a rat model. Tendon plasticity in response to skeletal muscle injury merits further investigation to understand its physiological relevance and potential clinical implications.

## Introduction

Acute and chronic tendon disorders, most commonly affecting the calcaneus tendon (CT), account for up to 50% of all sports injuries (Jarvinen et al., 2005b; Egger and Berkowitz, 2017). Tendons are fundamental biomechanical structures that connect muscles to bone to produce movement through force transmission (Wang et al., 2012). Tendons improve the economy of movement and amplify power output by their spring-like properties (Benjamin et al., 2008; Roberts and Azizi, 2010; Wilson and Lichtwark, 2011; Wang et al., 2012). Tendons also perform as mechanical buffers to protect skeletal muscle during contraction (Konow et al., 2012).

To perform this range of actions, tendons present abundant specialized extracellular matrix (ECM) resistant to tensile and compressive forces composed of approximately 70% of water by mass and collagen (mostly type I and III), proteoglycans, and other non-collagenous proteins organized in a hierarchical manner (Kjaer, 2004; Lavagnino et al., 2015). In addition, tendon contains a relatively small number of cells known as tenocytes arranged along collagen type I fibers forming an interconnected 3-dimensional network of cell and ECM. Importantly, the tenocytes transduce muscle-dependent loads to elicit functional remodeling of the ECM; tendons therefore demonstrate plasticity for dynamic adaptation in response to mechanical demands (Benjamin et al., 2008; Andarawis-Puri et al., 2015; Bohm et al., 2015; Lavagnino et al., 2015).

Homeostasis between tendon and muscle units requires a continuous bidirectional communication or cross-talk between tenocytes and muscle cells, known as myocytes (Schweitzer et al., 2010; Peng et al., 2017; Connizzo and Grodzinsky, 2018). Peng et al. (2017) reported that CT rupture and reconstruction led to “morphomechanical alterations” including a decrease of fascicle length, muscle thickness, and mechanical properties of the associated gastrocnemius muscle. This implies that tendon rupture and immobilization perturbed the bidirectional communication between tendon and muscle with functional consequences. On the other hand, Connizzo and Grodzinsky (2018) demonstrated that tendon can be affected by muscle using a mouse rotator cuff explant culture model which included the humeral head, supraspinatus tendon, and supraspinatus muscle. The authors found that pro-inflammatory cytokines from muscle and bone during stress deprivation culture, i.e., culture without mechanical stimuli, may cause tenocyte death after 3 days, which suggests a potential muscle-derived mechanism for onset of tendinopathy. Elsewhere, abnormal tendon loading such as underloading in cases of stress-shielding has been shown to induce an inflammatory response that weakens the structure and raise susceptibility to tendinopathies (Arnoczky et al., 2007; Cook and Purdam, 2009; Freedman et al., 2015; Bittencourt et al., 2016; Fouda et al., 2017). Such heterotypic interactions between tendon and skeletal muscle units may thus play a role in the etiology of tendinopathies and be relevant for clinical rehabilitation (Subramanian and Schilling, 2015).

Tendon morphogenesis is mediated by interactions with skeletal muscle *via* growth factors, protein migration, and force transmission (Freedman et al., 2015; Subramanian and Schilling, 2015). One of the major mechanosensitive pathways that control tendon responses to muscle-dependent loads during development and throughout life involves activation of latent transforming growth factor beta (*TGF-β*) in tendon ECM. This induces *SMAD* 2/3-dependent signal transduction and expression of transcription factors [e.g., mohawk homeobox (*MKX*), scleraxis (*SCX*), and *ERG1*] to induce collagen and proteoglycan synthesis (Subramanian and Schilling, 2015). Presumably, disruptions in muscle integrity as in the case of injury might lead to imbalances in these well-coordinated dynamic systems that in turn disrupt tendon integrity as well.

Although the cross-talk between tendon and muscle has been studied in recent years, to the best of our knowledge tendon plasticity in response to skeletal muscle injury has been neglected entirely. Thus, the aim of this study was to investigate the temporal effects of gastrocnemius muscle injury on the CT. We hypothesized that skeletal muscle injury would induce the CT to undergo maladaptive remodeling at the transcriptional level with concomitant harmful effects to morphology and biomechanical properties.

## Materials and Methods

### Animals and Experimental Groups

All procedures were conducted in accordance with the Guide for the Care and Use of Laboratory Animals (U.S. National Research Council, Washington D.C., USA). The research protocol received approval from the Animal Research Ethics Committee of the Catholic University of Brasilia, Brasília, Brazil (protocol number: 028/2015). Sixty-eight male Wistar rats (*Rattus norvegicus albinus*; weighing 300 +/− 25 g and 4 months of age at the start of experiments) were randomly divided into four experimental groups: control (C) group (animals not exposed to muscle injury) and 3, 14, and 28 days post gastrocnemius muscle (GA) injury (3D, 14D, and 28D). These time points were chosen to capture possible alterations in tendon during the three phases of the muscle regeneration process. Three days post-injure represents a degeneration/inflammation phase characterized by rupture and necrosis of the myofibers, formation of a hematoma, and inflammatory response. Fourteen days post-injure represents the remodeling phase characterized by maturation of regenerated myofibers with recovery of muscle function, fibrosis, and scar tissue formation. Twenty-eight days post-injure represents the complete or partial muscle maturation/functional repair. (Laumonier and Menetrey, 2016). The rats were placed in collective cages with members of the same group (3–4 animals/cage) with water and standard rodent chow (Purina^®^, Descalvado, São Paulo, Brazil) available *ad libitum* and exposed to 12 h light/dark cycles with temperature maintained at 20–22°C during the experiments.

### Muscle Injury (Cryolesion) Protocol

The rats were anesthetized with an intraperitoneal injection of xylazine (12 mg/kg of body weight) and ketamine (95 mg/kg of body weight) for the surgical procedures. To induce muscle injury in the medial and lateral belly of the GA, the skin around the muscle was first trichotomized and cleaned. A transversal skin incision (1 cm) over the muscle belly was then performed to expose the GA muscle. To induce cryo-lesions, a rectangular iron bar (length = 4 cm; width = 0.4 cm and height = 0.4 cm; total area = 6.56 cm^2^) was frozen in liquid nitrogen and then applied twice for 10 s to the GA muscle belly. The same procedure was repeated twice with a 30 s interval in between. After that, the skin was sutured using nylon thread 4–0 (Shalon Medical). This protocol is a common procedure and has already used in other studies (Miyabara et al., 2006; Oliveira et al., 2006; Durigan et al., 2008; Vieira Ramos et al., 2016) promoting muscle injury in TA muscle. This model induces a homogeneous injury in GA restricted to the surface region of the muscle belly which produces injuries similar to muscle contusion lesions (Jarvinen et al., 2005a).

### Muscle and Tendon Sample Collection

One at a time, the animals were euthanized using an intraperitoneal injection of xylazine solution (24 mg/kg of body weight) and ketamine (100 mg/kg of body weight) after the experimental periods and harvested. The CT was immediately dissected from posterior paws (3–8 min) and either; (1) frozen in RNase-free microtubes using liquid nitrogen (for qPCR) and then stored at −80°C; (2) fixed with 4% formaldehyde in (PBS, pH 7.4) for histological analysis; or (3) placed in physiological saline to prevent drying for mechanical testing. CT samples were approximately 8 mm in length and 1.5–2.5 mm in diameter.

### Muscle Histological Analysis

Serial histological cross-sections of the GA belly muscles were obtained (10 μm) using a cryostat microtome (Microm HE 505, Jena, Germany). The cross-sections were stained with hematoxylin and eosin (HE, Sigma Aldrich, St. Louis, MO, USA). Pictures of the cross-sections were acquired using an Olympus BV51 microscope equipped with an SV Micro Sound Vision digital camera (Preston South, Australia) at 10× magnification.

To confirm the presence of GA muscle injury in the cryo-lesion model, the signs characterized were: the presence of tissue infiltration with polymorphonuclear cells, disrupted and hypercontracted muscle fibers as well as centralized nuclei as previously reported (Oliveira et al., 2006; Durigan et al., 2008; Vieira Ramos et al., 2016). Posteriorly, one histological cross-section of each GA muscle located in the central region of muscle injury was choose to measure the total cross-sectional area, injury area, and uninjured area of the muscle using a software for morphometry (Image J 1.44p - National Institutes of Health, Bethesda, MD). To measure those areas, previously, pictures of the cross-section of each muscle were obtained by light microscopy to reconstruct the total muscle cross-section using a software PTGui Pro version 11.15. These procedures permitted us to identify and measure the injury and uninjured areas of the muscle for clarifying the injury × recovery time points.

### Calcaneus Tendon Histological Analysis

To evaluate morphological properties, the CT from 16 animals (4 per group) was fixed using 4% paraformaldehyde in phosphate-buffered saline (PBS) for 24 h at 4°C. After this step, they were washed in distilled water, serially dehydrated in ethanol, and embedded in paraffin.

Serial longitudinal sections (5 μm) of the peritendinous sheath and tendon proper were taken from the proximal and distal regions. The whole tendon was dissected out, and the longitudinal sectioning was cut (including proximal and distal regions) using glass knives and stained with hematoxylin and eosin (HE). This allowed investigation of adipose cells, blood vessels, and tendon cells from each region of the CT. Moreover, Masson’s trichrome staining was performed according to the standard procedures to examine the general appearance of collagen deposition (Woo et al., 2006; Tang et al., 2014). Pictures of the longitudinal-sections were acquired as described above. The proximal region of CT was determined by the myotendinous junction to its middle portion, and the distal region was determined by the middle portion to the CT enthesis.

### Image Analysis

For tendon H&E staining, 10 nonconsecutive digital images per region (peritendinous sheath and tendon proper, proximal and distal) were analyzed using Photoshop (Adobe Systems Inc., San Jose, CA, USA). A planimetry system using a translucent Weibel grid (Weibel, 1979) superimposed to each image was used to determine the volume density (Vv%) of adipose cells, blood vessels (lumen of blood vessel, endothelial cells, and perivascular sheath), other peritendinous sheath cells (includes all types of sheath cells, such as tenocytes, synovial cells, except adipose, and blood vessels cells), and tendon proper cells (fibroblasts and fibrochondrocyte-like cells). The stereology was performed by the point-counting method using coincidence of the grid points with the structures mentioned above. For the volume density estimation, the percentage of each structure in the peritendinous sheath and tendon proper was determined by multiplying the total number of grid points that coincide with the structures of interest by 100 and dividing by the total number of grid points falling on the peritendinous sheath or on the tendon proper.

ImageJ (National Institutes of Health, Bethesda, MD) was used in quantitative Masson’s trichrome analysis (Hernandez-Morera et al., 2016). Collagen fibers were quantified using these steps: image acquisition and processing, setting the scale, deconvolution of the color images, and quantification of the collagen fibers. The entire protocol was performed as described previously by Chen et al. (2018).

### RNA Extraction From TC Samples

Samples from 24 animals (6 per group) were homogenized in a tube containing five stainless steel balls (diameter, 2.3 mm) (BioSpec Products, Bartlesville, OK, USA) and three silicon-carbide sharp particles (1 mm) (BioSpec Products) by being shaken in a FastPrep-24 instrument (MP Biomedicals, Solon, OH, USA). To attain complete tissue homogenization, the shaking process was repeated seven times with ice cooling between each shaking step to help prevent RNA degradation. Total RNA was extracted according to the TRIzol method described by Chomczynski and Sacchi (1987). A NanoDrop^®^ spectrophotometer (ND-1000; NanoDrop Technologies Inc., Wilmington, DE, USA) was used to quantify RNA concentrations in each sample by determining the absorbance ratio of 260–280 nm. *TURBO DNA-freeKit* (Ambion – Life Technologies – 1907 M) was used for DNA digestion, according to the manufacturer’s recommendations.

### qRT-PCR Reverse Transcription

For the evaluation of CT gene expression, a total of 1 μg of RNA extracted from each tendon were converted into cDNA (final volume 20 μL) using *SuperScript^™^ VILO^™^ MasterMix* reverse transcriptase (Invitrogen-Cat. 11755-010) according to the manufacturer’s protocol. To perform reverse transcription, the samples were incubated at 25°C for 10 min, at 42°C for 60 min, and at 85°C for 5 min before being stored at −20°C in a freezer.

### Quantitative Real-Time Polymerase Chain Reaction (qRT-PCR)

qRT-PCR was performed using *TaqMan Universal PCR Master Mix* system (Applied Biosystems, CA, USA – Cat. 4304437). Ten microliters of GoTaq Probe qPCR Master Mix (Promega – A6102) were homogenized and combined with 1 μL of the primer 20×, an amount of cDNA determined according to the standard curve, and water for a final volume of 20 μL. The amplification reaction was performed by QuantStudio^™^ 3 (Applied Biossystems) according to the manufacturer’s instructions.

qRT-PCR was performed using a QuantStudio 3 Real-Time PCR System (Applied Biosystems) for the following genes: β-actin (*ACTB*), *ADAMTS-4*, biglycan (*BGN*), Type I collagen alpha 1 chain (*COL1A1*), Type III collagen alpha 1 chain (*COL3A1*), connective tissue growth factor (*CTGF*), decorin (*DCN*), *ERG1*, fibromodulin (*FMOD*), fibronectin (*FN*), glyceraldehyde 3-phosphate dehydrogenase (*GAPDH*), pro-insulin like growth factor IA (*IGF-1a*), interleukin-6 (*IL-6*), mohawk (*MKX*), matrix metalloproteinase-2 (*MMP-2*), ribosomal protein lateral stalk subunit P0 (*RPLP0*), scleraxis (*SCX*), Smad2 (*SMAD-2*), Smad3 (*SMAD-3*), syndecan-4 (*SDC-4*), transforming growth factor beta 1 (*TGF-β1*), tissue inhibitor of matrix metalloproteinase-1 (*TIMP-1*), tissue inhibitor of matrix metalloproteinase-2 (*TIMP-2*), tenascin C (*TNC*), tenomodulin (*TNMD*), and vascular endothelial growth factor (*VEGF*) (Table 1).

**Table 1:** List of tested genes.

mRNA	Code (life technologies)	mRNA	Code (life technologies)
*ACTB*	rn00667869	*MKX*	rn01755203
*ADAMTS-4*	rn02103282	*MMP-2*	rn01538170
*BGN*	rn01529736	*RPLP0*	rn03302271
*COL1A1*	rn01463848	*SCX*	rn01504576
*COL3A1*	rn01437681	*SMAD-2*	rn00569900
*CTGF*	rn01537279	*SMAD-3*	rn00565331
*DCN*	rn01503161	*SDC-4*	rn00561900
*ERG1*	rn00561138	*TGF-β1*	rn00572010
*FMOD*	rn00589918	*TIMP-1*	rn01430873
*FN*	rn00569575	*TIMP-2*	rn00573232
*GAPDH*	rn01775763	*TNC*	rn01454947
*IGF-1a*	rn00710306	*TNMD*	rn00574164
*IL-6*	rn01410330	*VEGF*	rn01511602

*β-actin (ACTB), ADAMTS-4, biglycan (BGN), Type I collagen alpha 1 chain (COL1A1), Type III collagen alpha 1 chain (COL3A1), connective tissue growth factor (CTGF), decorin (DCN), ERG1, fibromodulin (FMOD), fibronectin (FN), glyceraldehyde 3-phosphate dehydrogenase (GAPDH), pro-insulin like growth factor IA (IGF-1a), interleukin-6 (IL-6), mohawk (MKX), matrix metalloproteinase-2 (MMP-2), ribosomal protein lateral stalk subunit P0 (RPLP0), scleraxis (SCX), Smad2 (SMAD-2), Smad3 (SMAD-3), syndecan-4 (SDC-4), transforming growth factor beta 1 (TGF-β1), tissue inhibitor of matrix metalloproteinase-1 (TIMP-1), tissue inhibitor of matrix metalloproteinase-2 (TIMP-2), tenascin C (TNC), tenomodulin (TNMD), and vascular endothelial growth factor (VEGF)*.

For each gene, all samples were amplified simultaneously with technical duplicates from the same cDNA in a single run. The expression of each target gene was normalized based on the expression of the constitutive RPLPO gene, which was used as the control of endogenous RNA, due to lower intra and intergroup variability compared to the other housekeeping genes tested (*GAPDH* and β-actin). The ∆Ct values of the samples were determined by subtracting the mean Ct value of the target gene from the mean Ct value of the housekeeping gene. Subsequently, the ∆∆Ct values were calculated by subtracting the ΔCt value of the condition of interest from the ΔCt of the control condition. Finally, 2^−ΔΔCt^ values were computed for presentation of relative expression data.

It is important to highlight that the amount of sample and the efficiency of the reaction of each gene analyzed in the present study were determined from a standardization curve, having slope reference parameters equal to −3.3, *R*^2^ = 0.9–1.0 and efficiency above 90%. All data generated or analyzed in this study were displayed at a database repository Gene Expression Omnibus (GEO, NCBI) (Accession-GSE131884)^1^.

### Biomechanical Tests

The test length and cross-sectional area (CSA) of tendons were determined by measuring the width and thickness of three different parts of the test region using digital calipers (Digimess Instrumentos de Precisão Ltda., São Paulo, SP, Brazil) and multiplying their means before the test. The 28 tendons (7 per group) were fixed to an Instron 5500R test instrument by clamps attached to their proximal and distal regions. Custom-sized clamps wrapped with filter paper were used to protect the specimens from macroscopic damage and/or slipping off during testing (Probst et al., 2000). Each tendon was stretched to failure at a constant displacement rate of 1 mm/min using a 0.2 kN load cell (S2 transducer force – S2M/200N-1 – HBM, Inc., USA) (Tohyama and Yasuda, 2000). The data were used to construct force-displacement curves from which the stress-strain curves were derived. Based on these curves, the following parameters were calculated: displacement at maximum load (mm), maximum load (N), maximum stress (MPa), maximum strain (%), elastic modulus (MPa), energy to failure (N-mm), and CSA (mm^2^) according to de Cassia Marqueti et al. (2017) and Nakagaki et al. (2007). Each sample elastic modulus was calculated based on the slope of the linear portion of the stress-strain curve. All biomechanical tests for each time point were performed in a single day by a single operator.

### Statistical Analysis

The results from gene expression were presented according to Heinemeier et al. (2007b) and Marqueti et al. (2018). The Shapiro-Wilk test was applied to check for normality of variables, and Levene’s test was used to analyze homogeneity of variance. For the variables that did not have homogeneity of variance, the Welch test was applied to adjust the degrees of freedom of the residuals. Mean values were compared between groups by one-way ANOVA with the Bonferroni correction. In relation to the data that did not present normal distribution, the nonparametric Kruskal-Wallis test was applied. When appropriate pairwise comparisons were performed using Dunn’s procedure with Bonferroni correction for multiple comparisons, the level of significance was *p* ≤ 0.05. All analyses were conducted with statistical package for social sciences (SPSS, Inc., v. 21.0; IBM Corporation, Armonk, NY, USA) and GraphPad Prism 6.0 software (San Diego, California, USA). Stress-strain and force-displacement curves were generated using MATLAB (Release 2018A).

## Results

### Morphology of Muscle Lesions

**Figure 1:**
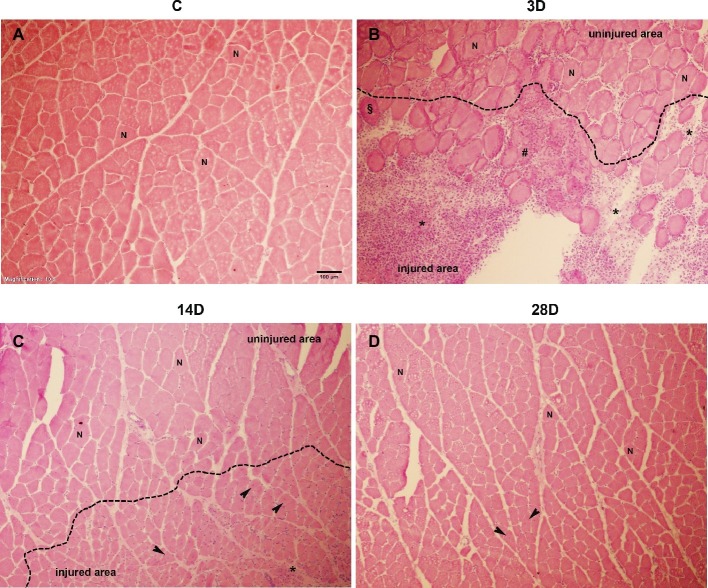
Histological sections of the muscle healing process of gastrocnemius muscle in four groups post-operation hematoxylin-eosin (HE) staining: **(A)**, control (C); **(B)**, 3 days post gastrocnemius muscle injury (3D); **(C)**, 14 days post gastrocnemius muscle injury (14D); **(D)**, 28 days post gastrocnemius muscle injury (28D). Inflammatory infiltrate (*), necrotic fiber (#), hypercontrated fibers (§); centralized nucleus (black arrow), and fibers with normal appearance (N). Magnification: 10×. The bar represents 100 μm.

In order to evaluate the presence of muscle injury after cryo-lesion, qualitative analysis of histological sections stained with HE was performed in all experimental groups (Figure 1). Cross-sectional area of GA muscle in C group showed intact muscle fibers, with no sign of injury (Figure 1A). In contrast, in 3D group post-injury, necrotic fibers, hypercontracted fibers, and inflammatory infiltrate, labeled in the figure by the flags [hash (#), paragraph (§), and asterisk (*), respectively], indicated the lesion was in the inflammatory phase of healing (Figure 1B). In the 14D group post-injury, centralized nuclei were visible (indicated in the figure by the black arrow), characterizing a regenerative process (Figure 1C). Finally, in the 28D group post-injury central nucleation had almost disappeared (a sign of regeneration in injured muscles) (Vieira Ramos et al., 2016), evidencing that muscle regeneration was nearing completion. In general, the characteristics described above confirm that cryo-lesion caused injury in the GA muscle.

### Animals Weight, Muscle Weight, and Injury and Uninjured Cross-Sectional Area of GA Muscle Middle Belly

We observed higher injury area in the 3D and 14 D groups when compared with the C group, respectively (*p* = 0.001; *p* = 0.001), besides the 14D group showed the lowest values when compared to the 3D group (*p* = 0.001). Additionally, the 28D group displayed lower injury area when compared with the 3D and 14D group, respectively (*p* = 0.001; *p* = 0.001). The total area was lower in the 14D group when compared with the C and 3D group, respectively (*p* = 0.001; *p* = 0.01; Table 2). No changes were observed in animals and muscle weight among the experimental groups (*p* > 0.05).

**Table 2:** Total and uninjured cross-sectional area of gastrocnemius muscle.

Groups	Animal weight (g)	Muscle weight (g)	Total area (mm^2^)	Injury area (mm^2^)	Injury area (%)
C	340.0 ± 23.85	1.66 ± 0.16	15.975 ± 1.542	0	0
3D	354.5 ± 34.6	1.67 ± 0.15	14.678 ± 2.303	1.773 ± 330.5^a^	12.1
14D	338.8 ± 22.2	1.56 ± 0.05	10.374 ± 1.141^a^^,^^b^	0.581 ± 0.13^a^^,^^b^	5.6
28D	361.5 ± 38.0	1.63 ± 0.15	12.484 ± 921.4^a^	0^b^^,^^c^	0

*The data are mean ± SD. The experimental groups: control (C), 3 days (3D), 14 days (14D), and 28 days post gastrocnemius muscle injury (28D)*.

a *Significantly different from C*.

b *Significantly different from 3D*.

c *Significantly different from 14D, p < 0.05. (n = 4 per group)*.

### Muscle Injury Alters Gene Expression in Rat Calcaneus Tendon

In order to analyze the GA lesion impact on CT, the expression of key genes that regulate the ECM in tendon homeostasis and remodeling was evaluated. Gene expression analysis was normalized by constitutive gene *RPLP0*. We also tested housekeeping genes *β-actin* and *GAPDH* but they were not stable; notably, *GAPDH* was the least stable gene expressed of all genes tested.

**Figure 2:**
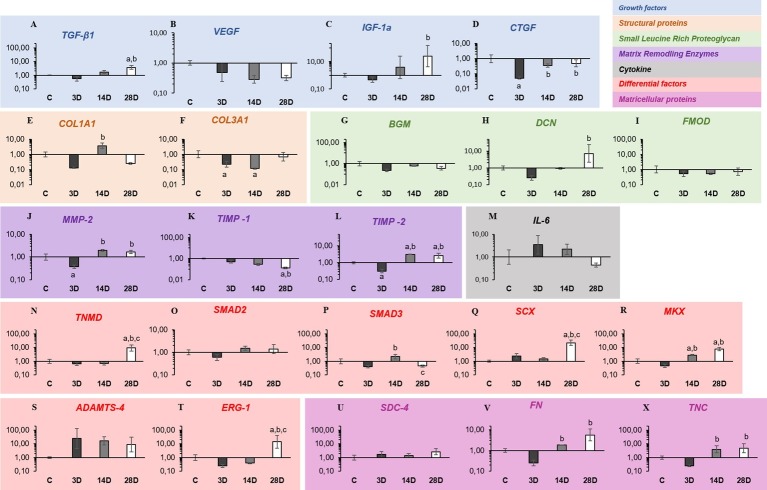
Gastrocnemius muscle injury modifies the gene expression related to ECM synthesis, remodeling, and tendon repair. **(A–D)** Growth factors (blue): transforming growth factor-beta 1 (*TGF-β1*), vascular endothelial growth factor (*VEGF*), pro-insulin like growth factor IA (*IGF-Ia*), and connective tissue growth factor (*CTGF*). **(E,F)** Structural proteins (light orange): collagen, type I, alpha 1 (*COL1A1*) and collagen, type III, alpha 1 (*COL3A1*). **(G,I)** Small leucine-rich proteoglycan (green): biglycan (*BGN*), decorin (*DCN*), and fibromodulin (*FMOD*). **(J–L)** Matrix remodeling enzymes (violet): matrix metalloproteinase-2 (*MMP-2*), tissue inhibitor of matrix metalloproteinases-1 (*TIMP-1*), and tissue inhibitor of matrix metalloproteinases-2 (*TIMP-2*). **(M)** Cytokine (gray): interleukin-6 (*IL-6*). **(N–T)** Differential factors (beige): tenomodulin (*TNMD*), Smad2 (*SMAD2*), Smad3 (*SMAD3*), scleraxis (*SCX*), mohawk (*MKX*), *ADAMTS-4*, and *ERG-1*. **(U–X)** Matricellular proteins (pink): Syndecan-4 (*SDC-4*), fibronectin (*FN*), and tenascin C (*TNC*). The experimental groups: control (C), 3 days post gastrocnemius muscle injury (3D), 14 days post gastrocnemius muscle injury (14D), 28 days post gastrocnemius muscle injury (28D). a, significantly different from C; b, significantly different from 3D; c, significantly different from 14D. *p* < 0.05 (n = 6 per group).

*Growth factors*: *TGF-β1* mRNA levels were upregulated in the 28D group when compared with C and 3D groups, respectively (*p* = 0.02; *p* = 0.001; Figure 2A). No changes were observed in mRNA levels of *VEGF* gene expression among the groups (*p* > 0.05; Figure 2B). *IGF-1a* mRNA levels were significantly upregulated in the 28D group when compared with the 3D group (*p* = 0.02; Figure 2C). *CTGF* mRNA levels were downregulated in the 3D group when compared with the C group (*p* = 0.001; Figure 2D), whereas the 14D and 28D groups demonstrated upregulation when compared with the 3D group (*p* = 0.02 and *p* = 0.01, respectively; Figure 2D).

*Structural proteins*: *COL1A1* mRNA levels were downregulated in the 3D group when compared with the C group (*p* = 0.03; Figure 2E). However, the 14D group showed upregulation when compared with the 3D group (*p* = 0.001; Figure 2E). Regarding *COL3A1*, the 14D group displayed upregulation in mRNA levels when compared with the C group (*p* = 0.02; Figure 2F).

*Small leucine rich proteoglycans (SLRP)*: *DCN* mRNA levels were significantly upregulated in the 28D group when compared with the 3D group (*p* = 0.001; Figure 2H). No changes were observed in mRNA levels of *BGN* and *FMOD* (*p* > 0.05; Figures 2G,I).

**Figure 3:**
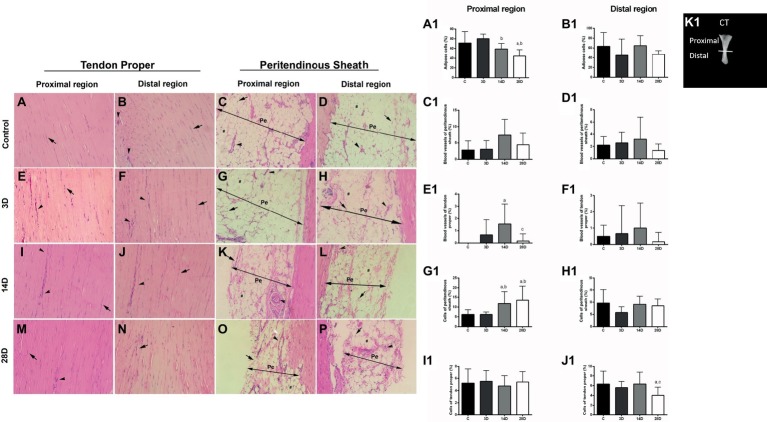
Morphological properties in calcaneal tendon are altered following gastrocnemius muscle injury. **(A1–J1)** express Vv% variation of structural elements found in the proximal and distal regions of the CT in each experimental group. **(A–P)** evidenced longitudinal sections (5 μm) of the proximal and distal regions of CT [tendon proper and peritendinous sheath (Pe) stained with hematoxylin-eosin]. Control group (C): tendon proper of the proximal region **(A)** and distal region **(B)** indicating cells (compact arrow) and vessels (arrowhead). Pe of proximal region **(C)** and distal **(D)** indicating cells (compact arrow), adipose cells (asterisk), and vessels (arrowhead); 3 days post gastrocnemius muscle injury (3D): tendon proper of the proximal region **(E)** and distal region **(F)** indicating cells (compact arrow) and vessels (arrowhead). Pe of the proximal **(G)** and distal **(H)** regions indicating cells (compact arrow), adipose (asterisks), and vessels (arrowhead); 14 days post gastrocnemius muscle injury (14D): tendon proper of the proximal region **(I)** and distal region **(J)** indicating cells (compact arrow) and vessels (arrowhead). Pe of the proximal **(K)** and distal **(L)** regions indicating cells (compact arrow), adipose (asterisks), and vessels (arrowhead); 28 days post gastrocnemius muscle injury (28D): tendon proper of the proximal region **(M)** and distal region **(N)** indicating cells (compact arrow) and vessels (arrowhead). Pe of the proximal **(O)** and distal **(P)** regions indicating cells (compact arrow), adipose (asterisks), and vessels (arrowhead). **(K1)** shows the dissected CT (adapted from Covizi et al., 2001) and indicates the position corresponding to proximal and distal regions. The data are mean ± SD. a = significantly different from C; b = significantly different from 3D; c = significantly different from 14D; *p* < 0.05. Magnification: 400×. The bar represents 40 μm. (*n* = 4 per group).

*Matrix remodeling enzymes*: *MMP-2* mRNA levels were upregulated in the 3D group when compared with the C group (*p* = 0.02; Figure 2J), whereas the 14D and 28D groups demonstrated upregulation when compared with the 3D group, respectively (*p* = 0.001; *p* = 0.001; Figure 2J). Additionally, *TIMP-1* mRNA levels were downregulated in the 28D group when compared with the C and 3D group, respectively (*p* = 0.001; *p* = 0.002; Figure 2K). On the other hand, *TIMP-2* mRNA levels were downregulated in the 3D group when compared with the C group (*p* = 0.01; Figure 2L); however, the 14D and 28D groups showed upregulation compared with the C (*p* = 0.01; *p* = 0.04 Figure 2L) and 3D groups, respectively (*p* = 0.001; *p* = 0.001; Figure 2L).

*Cytokine*: No changes were observed in mRNA levels of IL-6 among the groups (*p* > 0.05; Figure 2M).

*Developmental/progenitor and differentiation factors*: *TNMD* mRNA levels were significantly upregulated in the 28D group when compared with the C, 3D, and 14D groups, respectively (*p* = 0.01; *p* = 0.001; *p* = 0.001; Figure 2N). No changes were observed in mRNA levels of *SMAD-2* among the groups (*p* > 0.05; Figure 2O). However, *SMAD-3* mRNA levels were upregulated in the14D group when compared with the 3D group (*p* = 0.01; Figure 2P), but the 28D displayed downregulation when compared with the 14D group (*p* = 0.01; Figure 2P). *SCX* and *ERG-1* mRNA levels were significantly upregulated in the 28D group when compared with the C (*p* = 0.001; *p* = 0.02; Figures 2Q,T), 3D (p = 0.002; p = 0.001; Figures 2Q,T), and 14D groups, respectively (p = 0.001; p = 0.004; Figures 2Q,T). Moreover, *MKX* mRNA levels were upregulated in CT of the 14D and 28D groups when compared with the C (*p* = 0.04; *p* = 0.001; Figure 2R) and 3D groups, respectively (*p* = 0.001; *p* = 0.001; Figure 2R). No changes were observed in mRNA levels of *ADAMTS-4* among the groups (*p* > 0.05; Figure 2S).

*Matricellular proteins*: No changes were observed in mRNA levels of *SDC-4* among the experimental groups (*p* > 0.05). However, FN and TNC mRNA levels were significantly upregulated in the 14D and 28D groups when compared with the 3D group, respectively (*p* = 0.01; *p* = 0.01; *p* = 0.01; *p* = 0.01; Figures 2G,H,U,V,X).

### Light Microscopy of Tendon Hematoxylin and Eosin Staining

HE staining was performed to investigate adipose cells, blood vessels, peritendinous sheath cells, and tendon proper cells in proximal and distal regions of CT (Figures 3A-P, A1-J1). The 14D group presented lower adipose cell Vv% in the proximal region of the CT when compared with 3D group (*p* = 0.01; Figures 3K,A1). Moreover, the 28D group displayed decreased adipose cell Vv% when compared with the C and 3D groups, respectively (*p* = 0.001; *p* = 0.001; Figures 3O,B1). We also observed higher blood vessel Vv% in the proximal region of tendon proper in the 14D group when compared with the C group (*p* = 0.002; Figures 3I,E1); however, the 28D group showed lower blood vessel Vv% in the same region of tendon proper when compared with the 14D group (*p* = 0.01; Figures 3M,E1). Finally, 14D and 28D groups demonstrated increased peritendinous sheath cell Vv% in the proximal region when compared with the C (*p* = 0.04; *p* = 0.001; Figures 3K,O,G1) and 3D groups, respectively (*p* = 0.04; *p* = 0.001; Figures 3K,O,G1).

No significant differences were observed between the groups with respect to blood vessel Vv% of peritendinous sheath and tendon proper cell Vv% in the proximal region of the CT (*p* > 0.05; Figures 3C,I1).

On the other hand, the 28D group presented lower cell Vv% in the distal region of tendon proper of the CT when compared with the C and 14D groups, respectively (*p* = 0.04; *p* = 0.04; Figures 3N,J1). Lastly, no significant differences were observed between groups with respect to adipose cell Vv% and blood vessel Vv% of peritendinous sheath and in tendon proper and peritendinous sheath cell Vv% in the distal region of the CT (*p* > 0.05; Figures 3B1,D1,F1,H1).

### Light Microscopy of Tendon Masson’s Trichrome Staining

**Figure 4:**
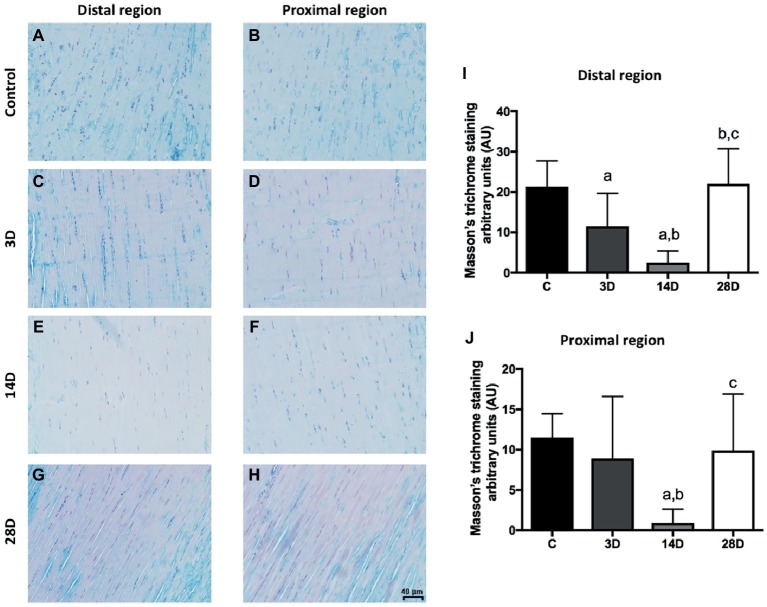
Masson’s trichrome staining indicated a lower collagen content of proximal and distal regions of the CT after gastrocnemius muscle injury. **(A–H)** evidenced longitudinal sections (5 μm) of the proximal and distal regions of CT (tendon proper). Collagen content (AU) in the proximal **(I)** and distal **(J)** regions. The data are mean ± SD. The experimental groups (*n* = 5): control (C), 3 days post gastrocnemius muscle injury (3D), 14 days post gastrocnemius muscle injury (14D), 28 days post gastrocnemius muscle injury (28D). a = significantly different from C; b = significantly different from 3D; c = significantly different from 14D; *p* < 0.05. Magnification: 400×. The bar represents 40 μm. (*n* = 4 per group).

Subsequently, the collagen content was analyzed in CT by Masson’s trichrome staining. Collagen content decreased in the distal region of the 3D group when compared with the C group (*p* = 0.02; Figures 4C,I). The 14D group displayed lower collagen content when compared with the C and 3D groups, respectively (*p* = 0.001; *p* = 0.03; Figures 4E,I). However, collagen content increased in the 28D group when compared with the 3D and 14D groups, respectively (*p* = 0.01; *p* = 0.001; Figures 4G,I).

With regard to the proximal region, collagen content decreased in the 14D group when compared with the C and 3D groups, respectively (*p* = 0.001; *p* = 0.01; Figures 4F,J), whereas collagen content increased in the 28D in comparison with 14D group (*p* = 0.01; Figures 4H,J).

### Tendon Biomechanical Properties Analysis

**Figure 5:**
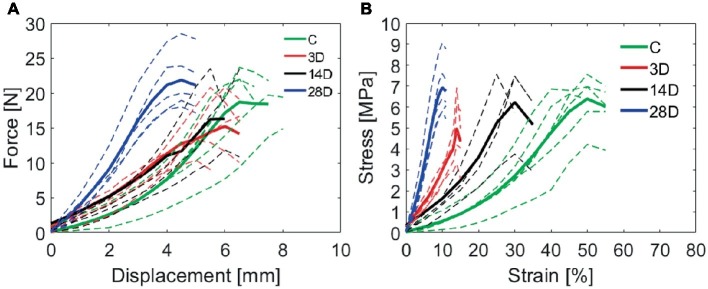
**(A)** Gastrocnemius muscle injury affects calcaneal tendon force-displacement curves. The experimental groups: control (C), 3 days (3D), 14 days (14D), and 28 days post gastrocnemius muscle injury (28D). **(B)** Calcaneal tendon stress-strain curves obtained after gastrocnemius muscle injury. Dotted lines indicate all the tested samples into each group and the thick line indicate the mean of the group. (*n* = 7 per group).

Figure 5 shows the force-displacement curves (A) obtained for the C, 3D, 14D, and 28D groups, from which the stress-strain curves were derived (Figure 5B). Of the six samples in each group that were subject to tensile testing, any that showed a sudden drop in force without breaking, i.e., evidence of slipping, on the force-displacement curves were excluded from the analysis: 0/6 from C; 1/6 from 3D; 2/6 from 14D; 1/6 from 28D. These samples otherwise, showed similar force-displacement profiles to those in their respective experimental groups; thus, the slipping was likely due to insufficient clamping of the samples rather than those samples being of particularly high-stiffness. For this reason, we do not believe that excluding them introduced a selection bias into the study.

Differences in the profile of stress-strain curves (Figure 5B), the 28D showed a greater capacity of resistance to tensional load despite of stiffnesses observed when compared with others groups. The analysis of the material properties was based on these curves and will be described below.

**Figure 6:**
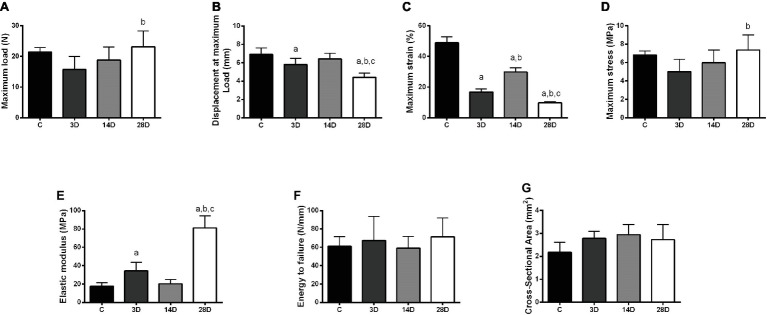
Biomechanical properties modifications in the calcaneal tendon in response to gastrocnemius muscle injury. Maximum load **(A)**, displacement at maximum load **(B)**, maximum stress **(C)**, maximum strain **(D)**, elastic modulus **(E)**, energy to failure **(F)**, and cross-sectional area **(G)** presented. The data are mean ± SD. The experimental groups: control (C), 3 days (3D), 14 days (14D), and 28 days post gastrocnemius muscle injury (28D). a = significantly different from C; b = significantly different from 3D; c = significantly different from 14D, *p* < 0.05. (*n* = 7 per group).

The maximum load in CT increased in 28D when compared with the 3D group (*p* = 0.02; Figure 6A). Regarding tendon extension (displacement at maximum load), the 3D group showed a lower maximum extension when compared to the control group (*p* = 0.04). In addition, 28D group decreases tendon extension compared to the other experimental groups (*p* = 0.02; Figure 6B). The maximum stress in tendon increased in 28D when compared with the 3D group (*p* = 0.02; Figure 6C).

**Figure 7:**
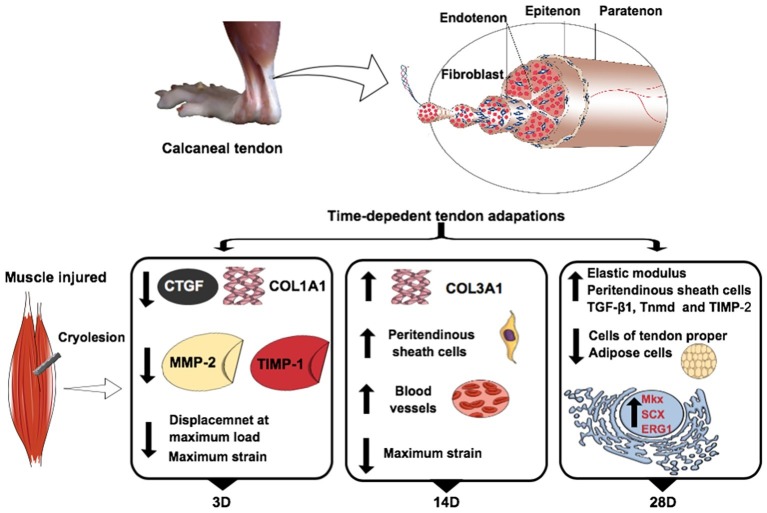
Skeletal muscle injury modulates calcaneal tendon plasticity. Main temporal analyses of the gene expression, morphological, and biomechanical properties of calcaneal tendon in relation to the control group. The experimental groups: control (C), 3 days (3D), 14 days (14D), and 28 days (28D) post gastrocnemius muscle injury. Connective tissue growth factor (*CTGF*), collagen, type I, alpha 1 (*COL1A1*), collagen, type III, alpha 1 (*COL3A1*), matrix metalloproteinase-2 (MMP-2), tissue inhibitor of matrix metalloproteinases-1 (*TIMP-1*), tissue inhibitor of matrix metalloproteinases-2 (*TIMP-2*), tenomodulin (*TNMD*), mohawk (*MKX*), scleraxis (*SCX*), and *ERG1*. The tendon calcaneal figure was adapted from Guzzoni et al. (2018).

The 3D group showed a decrease of maximum strain compared to control group (*p* = 0.001). However, the 14D group showed an increase of maximum strain compared to C and 3D groups, respectively (*p* = 0.001 and *p* = 0.001). Additionally, 28D displayed lower maximum stress when compared to the other experimental groups (*p* = 0.001; Figure 6D). Finally, 3D showed increased elastic modulus when compared with the control group and 28D groups showed the highest elastic modulus values when compared to the other groups (*p* = 0.016 and *p* = 0.001; Figure 6E). No significant differences were observed between groups with respect to energy to failure and cross-sectional area (*p* > 0.05; Figures 6F,G).

## Discussion

This study investigated the effects of skeletal muscle injury on time-course effects on the expression of key genes involved in tendon remodeling as well as the morphological and biomechanical properties of the calcaneal tendon (CT). For the first time, the present study demonstrated tendon plasticity in terms of gene expression as well as morphological properties and biomechanical adaptations in response to skeletal muscle injury. The morphological analysis was performed in the proximal and distal regions of CT. Although the proximal and distal regions are basely fibrous, the distal region of the CT is subjected to different levels of compressive forces and friction than the proximal region (Covizi et al., 2001). It was observed that distinct regions of CT differ in its response to muscle injury stimuli. Our new understanding of tendon plasticity in response to skeletal muscle injury may have crucial implications for treatment of tendinopathy.

### Tendon Response at Three Days Post Muscle Injury (3D)

The muscle showed the greatest disruption at 3D, indicating that the CT might also have been affected at this time point (Figure 1). Modulation in *CTGF* and *COL1A1* gene expression has been previously reported after tendon perturbations such as exercise (net collagen degradation after 24–36 h followed by net synthesis by up to 72 h) (Magnusson et al., 2010) and tendinopathies models (reduction in *COL1A1* both 24 and 72 h of glutamate exposure) (Dean et al., 2015). The novelty of our study was that skeletal muscle injury also decreases *CTGF* and *COL1A1* gene expression in tendon after 3 days. These results coincide with decreased collagen content in the distal tendon region at the same time, indicating what may be a progression toward a degenerative status. *CTGF* modulates many biological processes in tendon, including cell adhesion, migration, proliferation, and angiogenesis (Chiquet et al., 2003; Nakama et al., 2006; Liu et al., 2015b). Blockade of *CTGF* action or inhibition of *CTGF* synthesis may be associated with a decrease of collagen synthesis in fibroblast cellular culture (Duncan et al., 1999), which can cause detrimental effects on structural support, protection, and maintenance in tendon functional integrity. A plausible explanation for the downregulation of *CTGF* and *COL1A1* and decreased collagen content at 3D could be the lack of mechanical stimulus in tendon induced by muscle injury (i.e., stress shielding), since previous studies reported that mechanical stress can induce a high-level of *CTGF* expression and collagen I synthesis (Schild and Trueb, 2002; Chiquet et al., 2003; Heinemeier et al., 2007a; Galloway et al., 2013). These data suggest that collagen homeostasis is disrupted at 3D following muscle injury.

The ECM homeostasis is in part controlled by the balance of *MMPs* and *TIMPs* (Minkwitz et al., 2017); thus, the decrease of *MMP-2* and *TIMP-2* at 3D post injury suggests that the tendon was undergoing remodeling in response to the skeletal muscle injury. In addition, muscle injury likely hinders the normal molecular interactions between tendon and skeletal muscle such as *MMPs*, growth factors, and migration/proliferation of proteins that modulate tendon morphogenesis (Subramanian and Schilling, 2015; Thankam et al., 2016). Overall, muscle injury appeared to disrupt normal homeostatic molecular signaling in CT.

The disruption at the gene expression level at 3D in CT was associated with a disruption in biomechanical properties. Specifically, the maximum strain in tendons was significantly reduced by 2.9-fold at 3D stress-strain curve; further, the stress-strain curve appeared considerably steeper compared to the uninjured control. This demonstrates that just 3D of muscle injury was sufficient to induce considerable mechanical alterations in tendon with reduced ability to withstand strain. Previous studies have shown that tendon biomechanical properties depend upon small leucine-rich proteoglycans (SLRPs; decorin and biglycan in particular) (Robinson et al., 2017). SLRPs are the predominant proteoglycans in tendon with decorin accounting for around 80% of the total proteoglycan content of the tissue (Samiric et al., 2004). In the present study, there were downward trends in gene expression of both decorin and biglycan at 3D relative to the control.

The tendon ECM remodeling and biological function are intimately linked to mechanical load and muscle contraction intensity (Galloway et al., 2013). It has been demonstrated that external controlled load through exercise is a primary factor to promote tissue remodeling, which can lead to structural and functional improvements (Galloway et al., 2013). However, overloading above the tendon capacity is widely studied for their potential damaging factor, as well as the abolition of muscle load (Millar et al., 2017). The muscle injury performed in the present study showed a great traumatic process in the muscle. Experimental studies have demonstrated biomechanical changes in CT only after 4 weeks of complete stress shielding in calcaneus tendon (Ikoma et al., 2013; Kaux et al., 2013). Likewise, at least 5 weeks of high-power strength training protocols, focused on the triceps sural complex, have been shown useful in modifying mechanical variables (Kubo et al., 2007). This factor demonstrates that mechanical privacy (by the abrupt decrease of muscle activity) in the current study have been enough to generate significant mechanical alterations in the tendon.

### Fourteen Days Post Injury (14D)

Types I and III collagen showed variable expression over time. The upregulation of *COL1A1* post 14 days was associated with increased *MKX* and *SMAD3*, which are important regulators of ECM production including expression of *COL1A1* (Berthet et al., 2013; Liu et al., 2015a). In contrast, *COL3A1* showed a trend toward a decrease at 3D post-injury relative to the control followed by a further decrease at 14D. At the same time, Masson’s trichrome staining revealed lower collagen content at 14D relative to the control and the 3D post injury group. The decline in collagen content at 14D may thus be reflected in the shallower stress-strain curve at 14D relative to 3D. We might speculate that the increase of *COL1A1* gene expression at 14D post-muscle injury may reflect a compensatory mechanism, producing collagen in response to the altered mechanical properties at 3D.

These findings with respect to type I collagen may be supported by the abundance of peritendinous sheath cells found in the 14D post skeletal muscle injury group. These cells may have roles in restoring collagen content. In addition, the upregulation of *CTGF, FN, MMP-2, TIMP-2, SMAD3*, and *MKX* 14 days post injury in comparison to 3D, indicating that tendon undergoes adaptation following skeletal muscle injury. This may have clinical relevance in the prevention and treatment of musculoskeletal injuries (Benjamin et al., 2008; Bohm et al., 2015).

While blood vessels in the control group were limited to the peritendinous sheath, we found them trend toward an increase in abundance at 14D in the peritendinous sheath and penetrating the tendon proper. This may have provided the nutritional and metabolic support for the active ECM remodeling in the CT in response to skeletal muscle injury. However, we also observed a downward trend in *VEGF* gene expression in 3D and 14D compared to the control group. Further investigations are required to explain this contradictory result; it may suggest that other adjacent molecular pathways or post-translational regulation were involved in producing the observed vascularization.

### Twenty-Eight Days Post Injury (28D)

Twenty-eight days post skeletal muscle injury had increased peritendinous sheath cell content in the proximal region of tendon compared with the control group. Moreover, 28D showed similar collagen content to the control group, suggesting recovery from the loss of collagen observed at 14D. These aspects may be linked to increased *TGF-β1* expression observed at 28D. *TGF-β1* is an important factor in stimulating ECM growth, turnover, and remodeling, being a well-known promoter of tendon fibroblast proliferation and collagen synthesis (Kjaer, 2004; Gumucio et al., 2015; Goodier et al., 2016). *SCX*, *MKX*, and *ERG1*, which are important transcription factors during tendon development and repair, also appear to regulate expression of matrix molecules as *TNMD* which is consistent with the concurrent upregulation of all these genes at 28D (Murchison et al., 2007; Shukunami et al., 2016; Wu et al., 2017). As well, these genes are regulated by the *TGF-β* superfamily (Mendias et al., 2012; Subramanian and Schilling, 2015).

*SCX* regulates fibroblast proliferation and initiation of tenocyte differentiation in response to mechanical stimuli (Murchison et al., 2007). Previously, decreased *SCX* was shown to be related to removal of tendon load, whereas it increased dramatically in response to physiological loading (Mendias et al., 2012). For example, Murchison et al., 2007 reported that mice with loss *SCX* expression have a disorganized ECM and reduced collagen which compromise the power transmission capacity of tendon. Thus, these adaptations post 28 days skeletal muscle injury may be important to maintain joint movement, passive elastic response, and the restoration of tendon strength (Subramanian and Schilling, 2015). For their part, *MKX* and *ERG1* were shown to be involved in the maintenance of differentiated tenocytes (Wu et al., 2017).

*TNMD* is a tendon-specific gene marker, known to be important for tendon maturation (Docheva et al., 2005). *TNMD* plays a role in proliferation, differentiation, and migration of tendon progenitor cells (TSPCs) as well as endothelial cells (Shukunami et al., 2005, 2016). Our findings demonstrate that regulation of *SCX, MKX, ERG1, TGF-β1*, and *TNMD* are linked to increased muscle activity. This agrees with previous studies showing overexpression of *TGF-β, SCX*, and *TNMD* induced by mechanical loading.

Previous studies have found structural (i.e., collagen fibrils irregularly arranged and loose) and biomechanical changes (i.e., poor dynamic storage energy) in CT after few weeks of complete stress shielding in calcaneus tendon (44, 45). In respect to biomechanical properties following skeletal muscle injury, it appears that after 28D, the CT retains similar load-bearing capacity to the uninjured control. However, the CT appeared to lose flexibility at 28D, given that it had increased Young’s modulus compared to all time points and the least ability to withstand high levels of strain. The increase in Young’s modulus at 28D seemed to correlate with collagen synthesis and collagen fiber organization in this period. In a future study, it may be interesting to see if these changes in biomechanical properties persist several months after the skeletal muscle injury, and whether these changes can be linked to future susceptibility to tendon injury.

Another novelty of our study was that 28 days post GA injury showed decreased adipose cells in tendon when compared with the control group. Previous data have already shown that high amounts of adipose cells may indicate tendon degeneration and deleterious effects of aging (Benjamin et al., 2004). For example, two previous studies conducted by our research group demonstrated that aging in rats is linked to high levels of fatty acid-binding protein 4 (a protein linked to adipose cells) and increased adipose cells in CT (Barin et al., 2017; Marqueti et al., 2018). These factors may compromise tendon structure leading to weakness (Marqueti et al., 2018). The finding of decreased adipose cells 28 days post injury could thus be a positive sign indicating ongoing recovery from the imbalances suffered by the GA injury. Additionally, decreased adipose cells in 28D may have contributed to lower overall thickness of the peritendinous sheath (Figure 3).

Some limitations of the present study should be highlighted, such as the inability to identify the cell types contributing to mRNA levels since gene expression analysis was conducted on the whole tendon. Moreover, the lack of immunoblot assay of essential proteins and immunofluorescence would be relevant to clarify adjacent mechanisms involved in tendon remodeling. We further note that due to the practical necessity of testing all samples in a single day at each time point, there is a risk for batch effects from day-to-day in biomechanical data. To address this, we performed tests as consistently as possible by a single operator and present data from all samples along with the means (Figure 5). While we have no evidence that a batch effect of relevant size occurred, we state this limitation here and look forward to the results of future similar studies. We next intend to evaluate gene expression, morphology, and biomechanical properties of muscle over time following tendon injury in a similar model to the present study.

## Conclusion

In summary, our data demonstrate plasticity of the calcaneus tendon in response to cryolesion of the gastrocnemius muscle in rats. The results suggest that such an adverse muscle condition initiates a complex period of remodeling spanning at least 28 days. At 3 days following injury, we observed dysregulation of signaling pathways associated with collagen I and disrupted mechanical properties; at 14 days, there was reduced collagen content but increased invasion of blood vessels into the tendon proper and peritendinous sheath cells; and by 28 days, there was a dramatic rise in Young’s modulus and gene expression of transcription factors related to ECM synthesis, remodeling, and repair. This study highlights the importance of muscle-tendon interactions and provides insight into their underlying mechanisms. Our results suggest that tendon may be susceptible to tendinopathies following skeletal muscle injury; future human studies may be warranted to investigate this potential association.

## Data Availability

All data generated or analyzed in this study were displayed at a database repository Gene Expression Omnibus (GEO, NCBI) (Accession GSE131884) (https://www.ncbi.nlm.nih.gov/geo/query/acc.cgi?acc=GSE131884).

## Ethics Statement

The animal study was reviewed and approved by Animal Research Ethics Committee of the Catholic University of Brasilia, Brasília, Brazil (protocol number: 028/2015).

## Author Contributions

FB, JD, and RM conceived and planned the design of the experiments. FB, RM, CA, JA, and AD performed the experiment, analyzed the data, and designed the figures from biomechanical test. FB, GR, IS, AR, and RM performed the experiments, analyzed the data, and designed the figures of rest of experiments. FB, IS, GR, AS, JD, and RM interpreted the results and worked in the writing of manuscript. RM, OF, and JD involved in planning and supervised the work, also contributed to the design and implementation of the research. AS, AA, JD, OF, and RM provided critical feedback and analysis of the manuscript. All authors discussed the results and contributed to the final manuscript.

### Conflict of Interest Statement

The authors declare that the research was conducted in the absence of any commercial or financial relationships that could be construed as a potential conflict of interest.

## Footnotes

^1^https://www.ncbi.nlm.nih.gov/geo/query/acc.cgi?acc=GSE131884
